# Impact of hospitalisation on health-related quality of life in patients with chronic heart failure

**DOI:** 10.1186/s12955-020-01508-8

**Published:** 2020-08-03

**Authors:** Fernando Albuquerque de Almeida, Maiwenn J. Al, Ron Koymans, Jarno Riistama, Steffen Pauws, Johan L. Severens

**Affiliations:** 1 ESHPM – Erasmus School of Health Policy and Management, Erasmus University Rotterdam, Rotterdam, The Netherlands; 2 iMTA – Institute for Medical Technology Assessment, Erasmus University Rotterdam, Rotterdam, The Netherlands; 3 Professional Health Services and Solutions, Philips Research Europe, Eindhoven, The Netherlands; 4 Chronic Disease Management, Philips Research Europe, Eindhoven, The Netherlands

**Keywords:** Quality of life, Health-related quality of life, Utility, EQ-5D, Hospitalisation, Heart failure

## Abstract

**Background:**

Empirical identification of the direct impact of hospitalisation in the change in utility could provide an interpretation for some of the unexplained variance in quality of life responses in clinical practice and clinical trials and provide assistance to researchers in assessing the impact of a hospitalisation in the context of economic evaluations. This study had the goal of determining the impact of nonfatal hospitalisations on the quality of life of a cohort of patients previously diagnosed with heart failure by using their quality of life measurements before and after hospitalisation.

**Methods:**

The impact of hospitalisation on health-related quality of life was estimated by calculating the difference in utility measured using the EQ-5D-3L in patients that were hospitalised and had records of utility before and after hospitalisation. The variation in differences between the utilities pre and post hospitalisation was explained through two multiple linear regression models using (1) the individual patient characteristics and (2) the hospitalisation characteristics as explanatory variables.

**Results:**

The mean difference between health-related quality of life measurement pre and post hospitalisation was found to be 0.020 [95% CI: − 0.020, 0.059] when measured with the EQ-5D index, while there was a mean decrease of − 0.012 [95% CI: − 0.043, 0.020] in the utility measured with the visual analogue scale. Differences in utility variation according to the primary cause for hospitalisation were found. Regression models showed a statistically significant impact of body mass index and serum creatinine in the index utility differences and of serum creatinine for utilities measured with the visual analogue scale.

**Conclusions:**

Knowing the impact of hospitalisation on health-related quality of life is particularly relevant for informing cost-effectiveness studies designed to assess health technologies aimed at reducing hospital admissions. Through using patient-level data it was possible to estimate the variation in utilities before and after the average hospitalisation and for hospitalisations due to the most common causes for hospital admission. These estimates for (dis) utility could be used in the calculations of effectiveness on economic evaluations, especially when discrete event simulations are the employed modelling technique.

## Background

Heart failure (HF) is a condition characterized by typical symptoms (e.g. breathlessness, ankle swelling and fatigue) and signs (e.g. elevated jugular venous pressure, pulmonary crackles and peripheral oedema) caused by a structural and/or functional cardiac abnormality, resulting in a reduced cardiac output and/or elevated intracardiac pressures at rest or during stress [1]. HF is a major health concern associated with significant morbidity, mortality, and reduced quality of life for patients. From a medical perspective, the goals of managing patients with HF consist in improving their clinical status, functional capacity, and quality of life, preventing hospital admission, and reducing mortality [1, 2]. Understanding the relationship between all these goals of HF therapy is of vital importance for informing the development of clinical practice guidelines and for approving or recommending new therapeutic interventions for HF.

Previously published studies indicate that quality of life or health-related quality of life (QoL or HRQoL, respectively; henceforth used interchangeably) in patients with HF is greatly impaired when compared to the general population [3–5]. The New York Heart Association (NYHA) functional classification – a system for classifying patients according to the severity of their symptoms – has been shown to be a strong independent predictor of QoL for patients with HF [3]. However, since NYHA functional class is only assessed during clinical visits and provides a relatively simplistic way of classifying the extent of heart failure based on patients’ limitations during physical activity, the underlying determinants of reduced quality of life in patients with HF remain hardly distinguishable [6], particularly HF-related events that are expected to have an impact on patient utility (e.g. hospitalisation) [7].

Economic evaluations published in the literature have used an estimated disutility for hospitalisation equivalent to the decrease in utility between a particular NYHA class and the one immediately worse [8]. In view of the high incidence of (re) hospitalisation in patients suffering from HF, in absence of a robust method for calculating the (dis) utility resulting from a hospitalisation, it becomes essential to explore the relationship between hospitalisation and quality of life. From a theoretical viewpoint it may be assumed that there is a relationship between hospitalisation and utility, although there is insufficient or unclear reporting of evidence about the impact on utility caused by the hospitalisation of HF patients, both in the magnitude of the effect and the duration of this same effect [9].

Current practice in economic evaluations aimed at estimating quality-adjusted life years (QALYs) consists in measuring utility at specific points in time and linearly interpolate these values so that they reflect a larger time period for the subjects under analysis. In others words, using the QALY model as the measure of effectiveness in economic evaluations implies missing temporary changes in utility, particularly when these changes are due to disease-related events. For instance, when having access to two consecutive utility measurements with the same value – one before and other after a particular event –, using the QALY model leads to an implicit assumption that the utility of that same patient was constant throughout both time points and that the event that took place had no influence in the QoL of that patient, even though this assumption is unlikely to hold in an event such as a hospitalisation [10, 11]. In this sense, empirical identification of the direct impact of hospitalisation on the change in utility could provide an interpretation for some of the unexplained variance in QoL responses in clinical practice and clinical trials, as well as it may provide assistance to researchers in designing trials aimed at assessing patient-reported outcomes.

This study had the goal of determining the impact of nonfatal hospitalisations on the QoL of a cohort of patients previously diagnosed with heart failure by using their QoL measurements before and after hospitalisation.

## Methods

### Data

We used the data from the Trans-European Network-Home-Care Management System (TEN-HMS) trial for our study. This trial investigated the impact of using home telemonitoring, nurse telephone support (NTS), or usual care (UC) in hospital admissions, hospital days, and rates of mortality. Details of the inclusion and exclusion criteria, follow-up, and results of the study have been reported elsewhere [12]. In brief, patients who were ready for discharge or who were recently discharged after an hospital admission due to heart failure were evaluated for inclusion conditional on the permission by their primary care physician. Inclusion criteria for patients consisted of a hospital admission due to or complicated by worsening heart failure lasting more than 48 h within the last six weeks, persisting symptoms of heart failure, LV (left-ventricular) ejection fraction < 40%, LV end-diastolic dimension > 30 mm/m (height), and being medicated with furosemide at a dose ≥40 mg/day or equivalent (e.g., ≥1 mg of bumetanide or ≥ 10 mg of torasemide). In addition, patients should have at least one of the following indicators of further increase in risk: (1) unplanned cardiovascular admission lasting more than 48 h within the previous 2 years; (2) LV ejection fraction < 25%; or (3) treatment with furosemide at a dose of ≥100 mg/day or equivalent. Patients younger than 18 years of age who were considered incapable of complying with home telemonitoring or who were awaiting revascularisation, cardiac resynchronisation, or heart transplantation were excluded.

### Theoretical framework

The health-related quality of life in heart failure depends on the specific characteristics of a given patient, such as the disease status, gender, comorbidities, age, among others [5]. Events that may alter any of the aforementioned characteristics are expected to have an indirect impact on HRQoL. Because it results from a temporary deterioration of the health status of the patient or a permanent change in health status deriving from the progression of the disease, being hospitalised is expected to have an effect in HRQoL.

In practice, utility measurements of HF patients are taken periodically, during clinical visits to the physician, and they are not always performed when particular events related to disease progression take place (e.g. pulmonary embolism, tachyarrythmia, hospitalisation). Hence, while the global trend in HRQoL can be summarised, the specific impact of the event may be concealed, leaving many associations that can be hypothesised. For instance, in the period before the event, QoL may be decreasing as a result of a decline in the health status of the patient – which may in part explain hospitalisation –, but after the event QoL may improve again. As a result, the difference between the last utility measurement pre-event and the first measurement post-event could be zero or even show an increase in QoL. Alternatively, QoL may be stable when a very sudden decline in health triggers the event. After this event, QoL may improve again but it may not get back to the level it was before the decline happened. In this particular situation a decrease between the last pre-event utility measurement and the first post-event utility measurement would be recorded.

**Fig. 1:**
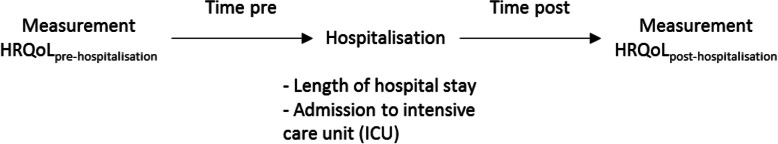
Schematic representation of HRQoL and its determinants

Using the data from the TEN-HMS trial we will try to answer our research question by analysing differences between QoL before and after hospitalisation. In this way we will be able to infer on the hypothesis that there is a difference in utilities resulting from the hospitalisation event. This approach entails that for every considered hospitalisation there is a period of time pre and post event that may vary for every observation and that may result in a different magnitude of the utility change between both measurements. Moreover, the particular characteristics of the hospitalisation – length of stay and whether the patient was admitted to the intensive care unit – are also likely to have an influence on the variation of HRQoL. Figure 1 provides a schematic representation of the framework that will be used for testing the hypothesis that hospitalisation impacts HRQoL and the determinants that may play a role in the measured variation.

### Measurement of the health-related quality of life

In the TEN-HMS trial health-related quality of life was measured using the three-level EQ-5D questionnaire (henceforth EQ-5D-3L), which consists of a descriptive system and a visual analogue scale (VAS) [13, 14]. The validity and reliability of the EQ-5D tool as an outcome measure within the cardiovascular area have been previously asserted [15]. More specifically, it has shown satisfying psychometric properties in cardiac rehabilitation [16].

For this study we calculated utilities by applying the utility weights previously identified for the Netherlands to the answers given to the EQ-5D-3L descriptive system questionnaire (utility values found this way will be referred to as index utilities, as opposed to VAS utilities) [17].

The EQ-5D-3L in the TEN-HMS trial was administered at baseline and it was repeated at 4, 8, 12, 16, 20, and 24 months, during scheduled clinical visits.

### Hospitalisation

There were sixty different causes for admission identified in the dataset. From these, hospitalisations could be classified into three major groups: (1) due to heart failure, (2) other cardiovascular, or (3) noncardiovascular. Information regarding the number of days spent in hospital, whether the patient was admitted to the ICU, and if the patient died during hospitalisation were also available from the data.

### Statistical analyses

#### HRQoL pre and post hospitalisation (base case)

In order to assess the impact of hospitalisation on HRQoL, we took the available EQ-5D-3L measurements immediately before and after hospitalisation. We then calculated the difference in utility measured for each individual patient – both using index and VAS utilities –, followed by the average utility difference for all patients who were hospitalised and had records for both measurements.

#### Sensitivity analyses

Four sensitivity analyses were performed. First, we excluded patients who experienced more than one hospitalisation between the EQ-5D-3L assessments of interest. Second, we only considered the hospitalisations for which the reason for admission was either heart failure or other cardiovascular event. Third, we stratified patients into consecutive groups for those who completed the EQ-5D-3L within X days of the non-fatal hospitalisation (for X = 20, 40, 60, 80, 100), in order to determine whether the time interval between the event and the subsequent HRQoL assessment had any effect on the magnitude of the utility change. And finally, we performed an analysis in which patients that died after hospitalisation and before completing the following HRQoL assessment were assigned a value of 0 for their utility measurement.

#### Utility variation by primary admission cause

In order to infer on the impact of the most frequent events that can lead to hospitalisation on utilities of HF patients, we used the methods from the base case analysis individually for each of the ten most common reasons for primary admission described in the dataset.

#### Impact of the characteristics of the patient and of the hospitalisation in the variation in HRQoL

We aimed at explaining the variation in differences between the utilities pre and post hospitalisation through two multiple linear regression models. The first used individual patient characteristics (measured at the same moment as utilities) as explanatory variables: body mass index, systolic and diastolic blood pressures, haemoglobin, serum sodium, and creatinine; the second used hospitalisation characteristics as the explanatory variables: length of hospital stay, number of days between the measurement before hospitalisation, number of days elapsed between the considered hospitalisation and the subsequent utility measurement, and a binary variable for the admission to the intensive care unit (ICU).

All statistical analyses were conducted using the programming language R [18].

## Results

### Baseline data

The demographic and clinical characteristics at baseline for the total population (*n* = 426) and the sub-population which has been hospitalised at least once can be found in Table 1. From the total population included in the study, 270 individuals (63.4%) experienced at least one hospitalisation (total number of hospitalisations = 583); the data from these patients were used in the analyses.

**Table 1:** Baseline Characteristics

Variable	Total population	Hospitalised population
Number	426	270
Hospitalised at least once (%)	270 (63.4)	270 (100)
Mean age, years (SD)	67.1 (13.1)	67.1 (13.2)
% patients age ≥ 70 years	48.1	48.5
% Women	22.5	19.6
Lives alone (%)	113 (26.5%)	69 (25.6%)
Lives with partner or friend (%)	313 (73.5%)	201 (74.4%)
Primary cause of heart failure (%)		
Coronary disease	254 (59.6%)	171 (63.3%)
Hypertension	27 (6.3%)	15 (5.6%)
Idiopathic dilated cardiomyopathy	95 (22.3%)	54 (20.0%)
Alcohol-related	11 (2.6%)	4 (1.5%)
Valve-related	28 (6.6%)	20 (7.4%)
Other	10 (2.3%)	6 (2.2%)
Comorbidities (%)		
Previous myocardial infarction	241 (57%)	163 (60%)
Valve disease/mitral regurgitation	156 (37%)/138 (32%)	101 (37%)/82 (30%)
Chronic or paroxysmal atrial fibrillation	192 (45%)	127 (47%)
Hypertension	200 (47%)	123 (46%)
Stroke, any	39 (9%)	29 (11%)
Chronic lung disease	103 (24%)	69 (26%)
Diabetes, any	149 (35%)	94 (35%)
Investigations (SD)		
Weight (kg)	76.7 (16.7)	77.1 (16.6)
Body mass index (kg/cm^2^)	26.2 (4.7)	26.3 (4.8)
Systolic blood pressure (mm Hg)	114.2 (19.3)	113.1 (19.7)
Diastolic blood pressure (mm Hg)	69.3 (11.3)	69.1 (11.3)
Hemoglobin (g/dl)	13.0 (2.1)	12.9 (2.0)
Serum sodium (mmol/l)	137.5 (5.0)	137.3 (5.1)
Serum creatinine (μmol/l)	138.7 (54.0)	143.7 (58.7)
Mean LVEF (%)	26.0 (7.5)	26.1 (7.7)
% with LVEF < 25%	50.2	47.4
NT proBNP (pg/ml), median [IQR]	365.5 [152.3 to 796.5]	393.0 [177.5 to 871.0]
Utility – Index (SD)	0.687 (0.242)	0.669 (0.246)
Utility – VAS (SD)	0.538 (0.192)	0.537 (0.189)

*Abbreviations*: *IRQ* interquartile range, *LVEF* left ventricular ejection fraction

The average age of included patients was 67.1 years old and there is a 4:1 ratio of men over women in this population. The great majority of patients have comorbidities, especially previous myocardial infarction and hypertension. Previous myocardial infarction is the main primary cause for HF in 63.3% of the cases, followed by idiopathic dilated cardiomyopathy (20.0%). Higher utilities at baseline (with higher standard deviation) were recorded for index utilities when compared to VAS utilities (0.669 ± 0.246 vs. 0.537 ± 0.189, respectively).

### Statistical analyses

#### HRQoL pre and post hospitalisation (base case)

**Fig. 2:**
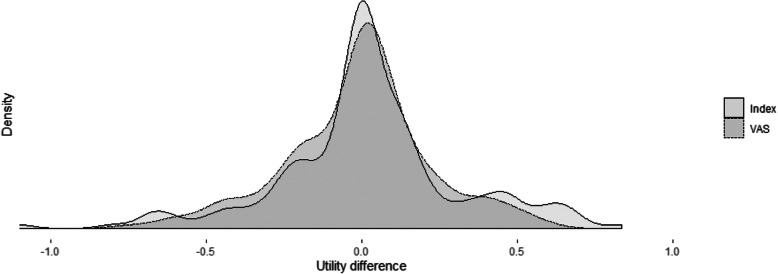
Distribution of the difference between measurements pre and post hospitalisation for index and VAS utilities

The mean difference between the HRQoL measurement pre and post hospitalisation was found to be 0.020 [95% CI: − 0.020, 0.059] for index utilities, and − 0.012 [95% CI: − 0.043, 0.020] for VAS utilities. There were no striking differences between the shape of the density curves of the utility variation when measured with either the EQ-5D-3L index or the VAS (cf. Figure 2).

#### Sensitivity analyses

**Fig. 3:**
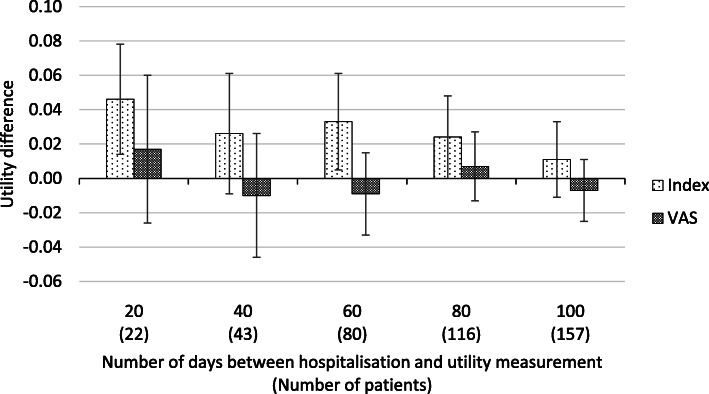
Stratification of patients according to number of days between hospitalisation and utility measurement

The first sensitivity analysis consisted of excluding patients who had more than one hospitalisation but did not die before the following HRQoL assessment. The analysis provided consistent results when compared to the primary analysis: utility variation of 0.000 [95% CI: − 0.081, 0.081] with index utilities and − 0.019 [95% CI: − 0.084, 0.046] with VAS. Secondly, restricting the analysis to hospitalisations that were due to cardiovascular conditions alone also does not change results of QoL variation substantially, with a calculated increase in utility of 0.023 [95% CI: − 0.016, 0.062] for index utilities and a decrease of − 0.009 [95% CI: − 0.041, 0.023] for VAS. Thirdly, stratifying patients according to the number of days elapsed between hospitalisation and the subsequent utility measurement, despite the large variance, shows that differences in utility measured with the VAS are noticeably smaller in absolute terms when compared to index utility differences (see Fig. 3). And finally, when assigning 0 to the utility score of patients who died after the hospitalisation, there was a significant decrease in the utilities pre and post hospitalisation of − 0.172 [95% CI: − 0.222, 0.122] with the EQ-5D index and − 0.133 [95% CI: − 0.171, − 0.096] for VAS.

#### Utility variation by primary admission cause

There were 456 records (78.22% of total number of hospitalisations) among the ten most common reasons for primary admission. The two causes with higher positive impact on the utility variation were respiratory/chest infection and ventricular tachycardia, whereas the causes with the highest negative impact on utilities were atrial fibrillation and myocardial infarction. Similarly to what was observed for the base case analysis, the calculated utility variations before and after hospitalisations for most of the primary admission causes registered high standard errors. The summarised results are presented in Table 2.

**Table 2:** Utility variation by primary admission cause

Primary admission cause	n (% total hospitalisations)	Utility variation (standard error)	
		Index	VAS
*Atrial Fibrillation*	12 (2.06)	−0.102 (0.213)	0.010 (0.184)
*Cardiovascular investigation*	13 (2.23)	0.031 (0.046)	−0.125 (0.250)
*Heart failure*	226 (38.77)	0.014 (0.069)	−0.024 (0.060)
*Myocardial Infarction*	14 (2.40)	−0.123 (0.075)	−0.175 (0.181)
*Other gastrointestinal cause*	25 (4.29)	0.070 (0.173)	−0.018 (0.105)
*Other not listed*	87 (14.91)	0.041 (0.093)	0.018 (0.076)
*Respiratory; Chest infection*	22 (3.77)	0.106 (0.186)	−0.062 (0.085)
*Stable Angina*	13 (2.23)	0.032 (0.215)	−0.148 (0.212)
*Unstable Angina*	31 (5.32)	−0.068 (0.106)	0.008 (0.157)
*Ventricular Tachycardia*	13 (2.23)	0.300 (0.334)	0.185 (0.219)
Total	**456 (78.22)**^a^		

^a^ From a total number of 583 hospitalisations among sixty admission causes

#### Impact of the characteristics of the patient and of the hospitalisation in the variation in HRQoL

The descriptive statistics of the patient characteristics measured during the quarterly clinical visits and the results of the regression models used to assess the impact of these characteristics on the differences in utility pre and post hospitalisation are presented in Table 3. The model showed a statistically significant impact (α = 0.05) of body mass index and serum creatinine in the index utility differences. For VAS utilities only serum creatinine was shown to explain the differences observed in statistically significant (α = 0.05) manner.

**Table 3:** Descriptive statistics of patient characteristics and their impact on utility pre and post hospitalisation

Variable	Descriptive statistics (mean [standard deviation])			Regression analysis Index			Regression analysis VAS		
	Pre hospitalisation	Post hospitalisation	Mean of Differences	Coefficient estimate	Standard error	*p*-value	Coefficient estimate	Standard error	*p*-value
Intercept	–	–	–	1.34 × 10^−2^	2.39 × 10^− 2^	0.5748	2.12 × 10^− 2^	1.87 × 10^− 2^	0.259
Body mass index (kg/cm^2^)	26.4 (5.9)	23.9 (9.9)	−2.5 (8.9)	1.73 × 10^− 2^	7.33 × 10^−3^	0.0198*	7.45 × 10^−4^	5.77 × 10^− 3^	0.897
Systolic blood pressure (mm Hg)	116.7 (21.3)	118.4 (22.6)	1.2 (21.5)	2.49 × 10^− 4^	1.39 × 10^− 3^	0.8579	−1.86 × 10^− 4^	1.07 × 10^− 3^	0.862
Diastolic blood pressure (mm Hg)	69.2 (10.9)	69.5 (11.5)	0.3 (14.2)	8.66 × 10^− 4^	2.24 × 10^− 3^	0.6991	2.01 × 10^− 4^	1.73 × 10^− 3^	0.908
Hemoglobin (g/dl)	8.8 (6.3)	8.8 (6.1)	−0.0 (2.5)	− 1.77 × 10^− 3^	9.38 × 10^− 3^	0.8510	− 1.10 × 10^− 3^	7.45 × 10^− 3^	0.882
Serum sodium (mmol/l)	134.6 (19.3)	133.4 (22.7)	−2.5 (26.5)	5.88 × 10^−5^	1.44 × 10^− 3^	0.9674	7.60 × 10^− 4^	1.13 × 10^− 3^	0.503
Serum creatinine (μmol/l)	136.6 (64.4)	139.5 (58.3)	1.1 (55.2)	−9.70 × 10^− 4^	4.52 × 10^− 4^	0.0339*	− 8.70 × 10^− 4^	3.55 × 10^− 4^	0.016*

* *p*-value < 0.05

Concerning the hospitalisation characteristics, none of these explanatory variables were shown to have a statistically significant (α = 0.05) relationship with the variation in utility for both index and VAS utilities. The results for this model are summarised in Table 4.

**Table 4:** Descriptive statistics of hospitalisation characteristics and their impact on utility variation pre and post hospitalisation

Variable	Descriptive statistics		Regression analysis Index			Regression analysis VAS		
	Mean	Standard deviation	Coefficient estimate	Standard error	*p*-value	Coefficient estimate	Standard error	*p*-value
Intercept	–	–	−9.11 × 10^− 3^	9.24 × 10^− 2^	0.922	9.93 × 10^− 2^	6.40 × 10^− 2^	0.122
# days before hospitalisation	52.7	39.0	− 3.02 × 10^− 4^	7.78 × 10^− 4^	0.698	6.56 × 10^− 4^	5.42 × 10^− 4^	0.227
# days after hospitalisation	74.8	41.5	3.23 × 10^− 4^	7.67 × 10^− 4^	0.674	−9.15 × 10^− 4^	5.21 × 10^− 4^	0.081
Length of stay (days)	13.7	24.9	−1.30 × 10^− 3^	1.64 × 10^− 3^	0.429	−1.2 × 10^− 3^	1.23 × 10^− 3^	0.309
Intensive care (%)^a^	5.69	–	7.76 × 10^− 2^	1.01 × 10^− 1^	0.445	1.24 × 10^− 1^	7.63 × 10^− 2^	0.107

^a^ dummy variable

## Discussion

Quantifying the impact of hospitalisation on QoL is particularly relevant for informing cost-effectiveness studies designed to assess health technologies primarily aimed at reducing admissions, especially when compared to technologies aimed at reducing the decline of the patient health status. To the best of our knowledge, this is one of the first studies to address the impact of hospitalisation in HRQoL of heart failure patients. In view of readmission being a common event for these patients [19, 20] – with the first few weeks after discharge from hospital being the highest risk period; between 20 and 30% of patients are readmitted within 30 days, rising to 50% at 6 months [21] –, it seemed relevant to have an estimate of the impact of this event on the HRQoL of HF patients, thereby overcoming the use of utility decrement estimates based on the assumption that patients progress to the immediately worse NYHA class after hospitalisation [8]. Using patient-level data we have calculated an empirical estimate for the difference between HRQoL before and after hospitalisation.

In this study we found a slight difference between the HRQoL measured before and after a hospitalisation: an increase in index utilities of 0.020 [95% CI: − 0.020, 0.059] and a decrease of − 0.012 [95% CI: − 0.043, 0.020] for VAS utilities. Even though there is a discrepancy between the directions of this change, the small magnitude of the effect – further substantiated by the relatively large confidence interval around the mean and the similarity between the density curves of the two methods – indicates that there is no significant evidence of a difference between utility pre and post hospitalisation when using either of the utility elicitation methods. Nonetheless, there are two possible explanations for the difference between index and VAS utilities: (i) VAS utilities tend to be lower than index utilities for the same individuals (see Table 1); and (ii) changes in index utilities measured with the EQ-5D-3L are prone to “jumps”, as they are only possible through a change in the patient self-assessment of his/her health state within the three possible levels – no problems, some problems, and extreme problems – for each of the five health dimensions. The five level EQ-5D (EQ-5D-5L) has since been introduced and it has proven to be a superior tool than EQ-5D-3L with respect to various measurement properties, enabling improvements in sensitivity and precision in health status measurement and the resulting utilities [22].

The findings for the base case analysis are further substantiated by the sensitivity analyses, except for the one that consisted of attributing 0 to the value of the utility post hospitalisation in patients who either died in hospital or before having an available measurement after the event. This analysis resulted in a decrease of − 0.172 [95% CI: − 0.222, 0.122] for the index utility and − 0.133 [95% CI: − 0.171, − 0.096] for the VAS utility.

However, it is crucial to note that hospitalisations in heart failure patients are heterogeneous and, therefore, the impact of these hospitalisations on QoL is likely to depend on the underlying clinical cause for admission. For instance, a hospitalisation resulting from a temporary deterioration in the health of a patient, typical in revolving-door patients, may lead to an improvement in QoL measured before and after hospitalisation, whereas a stroke or other disabling event is likely to show the opposite. Further, it may be difficult to attribute hospitalisation to a single cause or to a single disease factor in a disease like heart failure, especially when considering all the comorbidities that are frequently associated with the disease. The small effect encountered for the base case analysis might be due to the offset of hospitalisations caused by different underlying problems in HF patients.

The results found when analysing the HRQoL pre and post hospitalisation by primary cause for hospital admission seem to suggest that it is possible to distinguish the impact on QoL for different types of hospitalisation (see Table 2). In that analysis, hospitalisations due to respiratory/chest infection and ventricular tachycardia showed an improvement in QoL when considering the index utilities measured before and after admission, while hospital admissions attributed to atrial fibrillation and myocardial infarction showed a negative variation in index utilities measured before and after admission. These results appear to be in line with the hypothesis postulated in the previous paragraph.

The regression analyses for explaining the observed variation in utilities before and after hospitalisation were inconclusive concerning the characteristics of the hospitalisation. However, the difference in body mass index (only for index utilities) and serum creatinine (both for index and VAS utilities) pre and post hospitalisation showed a significant effect on the utility variation, albeit no informed explanation for the mechanism of this effect can be provided, as it was not covered by the scope of this study.

Although international guidelines are clear in prioritising quality of life in the management of patients with HF [1, 2], their perception on their quality of life is not always prone to a straightforward assessment in a trial setting [23]. Bosworth et al. [24] showed that psychosocial aspects and patient uncertainty about their prognosis are important components of quality of life among HF patients. Similarly, Heo et al. [25] found quality of life in patients with HF to be a multidimensional, subjective concept, affected by a variety of factors that do not only reflect HF symptoms and limitations in their daily life due to those symptoms, but also their active pursuit of happiness and relationships with others. Other factors such as anxiety, general distress, or depression have been shown to decrease QoL amongst HF patients [26, 27], whilst interventions aimed at improving patient self-care proved to have positive impact on QoL [28]. Following on these thoughts, it can be argued that hospitalisation is a source of distress for patients, who would therefore experience a decrease in HRQoL. In fact, a study by Harrison et al. [29] showed significant improvements in HRQoL associated with lesser use of emergency rooms – even though one can also argue on inverse causality, i.e. that fewer visits to the emergency rooms may be due to better health and thus higher HRQoL. Another study, by Lewis et al. [7], found that myocardial infarction survivors experiencing a nonfatal cardiovascular event (hospitalisation for heart failure, recurrent myocardial infarction, stroke, or sudden death/cardiac arrest) had a significant worsening of their HRQoL when compared to the ones who did not experience such event, suggesting that reducing nonfatal cardiovascular events might affect longitudinal changes in HRQoL.

Having an accurate estimate of the utility variation attributed to hospitalisation in HF patients would be a great addition to the economic evaluation arsenal. In fact, there are discrete event simulation models published in the cost-effectiveness literature that use “hospitalisation” as an “event” [30]. Especially for these cases, a good estimate of the (dis) utility of a hospitalisation would be of great value.

### Limitations

The variation between utilities pre and post hospitalisation showed a different magnitude from what was hypothesised and the value found for that variation was surrounded by a lot of uncertainty. There are some possible explanations that can be identified in the scope of the limitations of this study.

First, the patient population included in the analysis was already in a very advanced stage of the disease: (1) coping with chronic disease has been described to have a positive influence in the QoL perception of the ill patient [31] and (2) the fact that these patients have been previously hospitalised – as this was one of the inclusion criteria of the trial – may desensitise them to subsequent hospitalisations. Secondly, the measurement of HRQoL is not done at a particular moment related to the hospitalisation; in order to be comparable, the utility measurement should be done at admission, discharge, or, preferably, both – the mean number of days before the HRQoL measurement before hospitalisation is 52.7 and after hospitalisation is 74.8; both with large standard deviations (cf. Table 4). The results of the third sensitivity analysis should also be discussed in this context: in spite of the lack of statistical significance, results in Fig. 3 seem to suggest that the magnitude of the utility difference is higher when the HRQoL is measured closer to the hospitalisation date. Thirdly, we did not have information that would allow for adjusting for other factors that might have affected changes in HRQoL, including changes in medications and/or any surgical procedures done during hospitalisation. And finally, the EQ-5D-3L is an utility measurement tool that assesses global health status and that may not be as responsive as a disease-specific instrument like the Minnesota living with heart failure questionnaire (MLHFQ) [32].

### Recommendations for future research

Paying attention to the main issues that have been discussed so far, a few points should be stressed.

Some standardisation regarding the moments at which HRQoL is measured is desirable. This concept should be applicable not only in a controlled setting but also in current clinical practice. Conducting EQ-5D questionnaires or using other tools for measuring utilities may generate data that could turn out to be important in the development of guidelines for the management of heart failure. Special attention should be paid to the variance observed in HRQoL from clinical trials and the clinical practice. Some possible explanations for this variance are: (1) diseases with multiple comorbidities, where the trial population is often not representative of the real patient population, and (2) the Hawthorne effect, i.e. the mere attention paid by clinical trial personnel to study subjects, which may have beneficial effects on the QoL of participating trial patients [33, 34].

In the particular case of home telemonitoring – from which the population in this study originated – daily measurements of HRQoL could be performed. Considering most telemonitoring settings it is not expected that these measurements would constitute an increased burden for patients. Yet, the generation of longitudinal utility data would allow for investigating QoL as a predictor for hospitalisation. Health deterioration could be captured by trends in the data regarding patient-reported HRQoL. The analysis of these data could potentially result in the development of clinical decision rules or diagnostic algorithms that could avoid unnecessary hospitalisations, leading to potential cost savings and better health outcomes in the management of heart failure.

Considering that disease-specific instruments for HF (e.g. MLHFQ) can be more informative on patient perceived health status, a formal link between the outcomes of these questionnaires and a measure of utility should be established. In this way researchers could have access to more accurate information on patient-reported outcomes without compromising utility measurements that are normally used for economic evaluations.

And finally, HF-related research should focus on the determinants of HRQoL in heart failure patients. Although NYHA is a widespread classification of the severity of HF symptoms, the current capabilities for data collection and data generation are immense. They should be explored in order to open up possibilities for new classifications that could better suit the need of efficient management of heart failure patients.

## Conclusions

Knowing the impact of hospitalisation on health-related quality of life is particularly relevant for informing cost-effectiveness studies designed to assess health technologies aimed at reducing hospital admissions. Through using patient-level data it was possible to estimate the variation in utilities before and after the average hospitalisation and for hospitalisations due to the most common causes for hospital admission. These estimates for (dis) utility could be used in the calculations of effectiveness on economic evaluations, especially when discrete event simulations are the employed modelling technique.

## Data Availability

Not applicable.

## Abbreviations

HF: Heart failure
QoL: Quality of life
HRQoL: Health-related quality of life
NYHA: New York Heart Association
QALY: Quality-adjusted life year
TEN-HMS: Trans-European Network-Home-Care Management System
NTS: Nurse telephone support
UC: Usual care
LV: Left-ventricular
VAS: Visual analogue scale
ICU: Intensive care unit
MLHFQ: Minnesota living with heart failure questionnaire
